# The ART of resilience: a theoretical bridge across resilience perspectives

**DOI:** 10.3389/fpsyg.2025.1556047

**Published:** 2025-03-31

**Authors:** Moshe U. Farchi, Maya Peled-Avram

**Affiliations:** Department of Social Work, Research Center for Innovation in Social Work, Tel-Hai Academic College, Tel Hai, Israel

**Keywords:** resilience, coping resources, positive psychology, neuro psychology, heart rate variability

## Abstract

The theoretical understanding of psychological resilience has evolved significantly over recent decades, leading to diverse conceptual frameworks that emphasize different aspects of resilient adaptation. Some frameworks focus on resilience as a personal trait, others view it as a dynamic process, while still others emphasize the role of environmental and systemic factors. This theoretical paper introduces the ART framework (Acknowledgment, Reframe, and Tailoring), which provides an integrative perspective that bridges these seemingly disparate approaches. The ART framework offers a comprehensive understanding of how various resilience mechanisms work together in real-world contexts by focusing on the dynamic interplay between resource identification, reframing threats as challenges, and adaptive tailoring between resources and challenges. The ART framework incorporates and extends existing theoretical perspectives while providing a practical structure for understanding resilience development and intervention.

## Introduction

The Evolution of Resilience Theory The study of psychological resilience has undergone several paradigm shifts since its inception. Resilience, as a theoretical concept, has been extensively researched from diverse perspectives (Joyce et al., 2018), leading to multiple definitions and conceptualizations and, consequently, variation in methods and findings (Denckla et al., 2020). Despite discrepancies and scholarly debates (Ayed et al., 2019), most conceptualizations indicate that resilience involves exposure to significant adversity—ranging from ongoing daily hassles to major life events—and the manifestation of positive adaptation (Stainton et al., 2019). Resilience is often defined as the capacity to “bounce back” from challenging circumstances. It is considered the common response to adversity, as opposed to other trajectories, like recovery, characterized by a gradual return to baseline adjustment (Bonanno and Diminich, 2013).

Early studies focused on attributes that facilitate adaptation and the processes enabling it. This was followed by an expansion of the concept beyond the individual level, encompassing multiple social-ecological environments and resilience processes across space and time (Masten et al., 2021). As a characteristic, resilience refers to the constellation of personal and social resources an individual possesses that enable adaptation to adversity (Ayed et al., 2019). This conceptualization raises the trait versus state question: Is resilience a trait—heritable, stable over time, with distinctive personality qualities like the Big Five or a state, representing a flexible human potential like efficacy and hope, adaptable to social-ecological contexts (Luthans et al., 2007). The trait-based perspective has identified personal qualities—like hardiness, optimism, and self-efficacy—as key components of resilient functioning (Connor and Davidson, 2003). Prince-Embury (2014) suggested that the main characteristics leading to resilient outcomes include intellectual ability, an easy temperament, autonomy, self-reliance, communication skills, and effective coping strategies. Additionally, positive psychology—defined as the study of positive subjective experience (Csikszentmihalyi et al., 2014)—introduced a strengths-based approach, emphasizing virtues like optimism and self-esteem as pivotal in coping with adversity. This shift accentuated personal strengths and virtues in fostering resilience (Hogan, 2020).

From a developmental perspective, a prominent personal characteristic related to resilience is the quality of attachment (Holmes, 2017). The literature highlights early life experiences and attachment in shaping resilience, emphasizing the significance of secure attachment in overcoming adversity and promoting positive adaptation—cornerstones of resilience (Rasmussen et al., 2019). Internal and external factors (e.g., affect regulation and stable relationships), shaped by early caregiver experiences, also play a significant role in resilience development (Atwool, 2006). Studies increasingly demonstrate that resilient outcomes depend on complex interactions between individuals and their environments (Masten and Cicchetti, 2016). This understanding has led to process-oriented perspectives emphasizing the dynamic nature of resilience development and expression (Bonanno, 2004).

As a dynamic process, resilience may vary across situations and time (Curtis and Cicchetti, 2007). Researchers have identified different trajectories of post-adversity response. For example, Bonanno et al. (2015) differentiate between emergent resilience following chronic aversive events and minimal-impact resilience following acute events. From the process perspective, individuals develop and utilize assets and resources that promote positive outcomes in adversity (Métais et al., 2022). An asset is a personality characteristic, while a resource is external to the individual. This distinction reflects the complex interaction between individual and environment underlying the resilience process (Fergus and Zimmerman, 2005). Assets and resources, and risk factors hindering positive adjustment, constitute compensatory resilience processes (Métais et al., 2022). This social-ecological perspective views individual and environmental qualities transactionally, offering a more consensual resilience conceptualization (Kuldas and Foody, 2022). In this view, environmental factors, social supports, and cultural contexts are crucial in shaping resilient outcomes (Ungar, 2013).

At the neurobiological level, the hippocampus—critical for memory consolidation—helps integrate past experiences to inform future coping strategies (Davidson and McEwen, 2012). In terms of physiology, heart rate variability (HRV) indicates autonomic nervous system regulation; higher HRV is linked to greater emotional resilience and cognitive adaptability (Shaffer et al., 2014). The Need for Integration While each theoretical perspective has contributed valuable insights to our understanding of resilience, the field lacks a framework that integrates these diverse approaches while preserving their unique contributions.

The trait-based perspective highlights individual differences in resilience capacity, while the process-oriented approach emphasizes its dynamic and evolving nature. In addition, neuroscientific research addresses physiological regulation, and ecological models underscore the importance of environmental and contextual factors.

However, a unifying structure is needed—one that explains how resilience emerges, is sustained, and strengthened over time, while clarifying the mechanisms through which individuals and systems build and maintain resilience (Southwick et al., 2014). To address this need, resilience training programs have been developed for multiple populations, aiming to enhance coping capacities before, during, and after adversity (Chmitorz et al., 2018). These interventions vary in timing and focus but often remain grounded in partial frameworks.

In this context, the ART framework (Acknowledgment, Reframe, Tailoring) offers a cohesive model that synthesizes cognitive, emotional, social, and physiological mechanisms into a unified, actionable structure. It offers a practical response to the conceptual fragmentation in the field and provides structure for both theory and application, enhancing ability to understand and promote resilience across levels and settings.

## The ART of resilience

### Acknowledgment of coping resources

Acknowledgment is the first step in resilience, involving recognition of available coping resources and acceptance of the reality of the situation. From a psychological perspective, this aligns with acceptance-based coping strategies, shown to reduce distress by preventing emotional suppression and cognitive avoidance (Bonanno, 2004). Resilient individuals acknowledge adversity without becoming overwhelmed—a process that balances emotional awareness with problem-focused coping.

At the neuropsychological level, acknowledgment requires activation of the prefrontal cortex (PFC), which regulates emotional responses by modulating amygdala-driven stress reactions. The hippocampus, responsible for retrieving past experiences, connects present stressors with previously learned coping mechanisms (Davidson and McEwen, 2012). When individuals fail to acknowledge stressors, overactivation of the amygdala can lead to heightened sympathetic nervous system (SNS) responses, prolonged cortisol secretion, and impaired decision-making. In contrast, individuals with greater vagal tone and heart rate variability (HRV) exhibit better emotional regulation and cognitive flexibility (Lehrer and Gevirtz, 2014).

Self-efficacy (Bandura, 1997) is crucial in acknowledgment—individuals who believe in their ability to handle stressors tend to engage in proactive coping strategies. Interventions like awareness training and guided reflection exercises can enhance ability to recognize resources in times of distress. This is relevant in high-risk professions and among trauma-exposed individuals, where rapid recognition of coping mechanisms impacts mental health outcomes. By integrating cognitive, physiological, and psychological factors, acknowledgment lays the foundation for effective resilience.

### Reframe threats into challenges

Reframing is a core resilience strategy, allowing individuals to reinterpret stressors in ways that promote adaptive responses rather than avoidance or helplessness. This concept is deeply embedded in cognitive-behavioral therapy (CBT), through cognitive restructuring— identifying, challenging, and replacing maladaptive thoughts with more constructive perspectives (Zlomke and Jeter, 2020). Research suggests that optimistic cognitive styles relate to lower PTSD symptoms and greater post-traumatic growth (PTG), reinforcing reframing in psychological adaptation (Tedeschi and Calhoun, 2004).

From a neurobiological perspective, reframing is driven by PFC activation, which regulates emotional responses and modulates amygdala-driven fear reactions. Dopaminergic activity in the PFC enhances cognitive flexibility, allowing individuals to reinterpret distressing events more adaptively (Di Domenico and Mapelli, 2023). Studies show individuals with stronger PFC engagement exhibit better emotion regulation, lower PTSD risk, and improved problem-solving under stress (Felmingham et al., 2014). Additionally, higher HRV is associated with better stress regulation, creating a feedback loop whereby successful reframing enhances autonomic flexibility over time (Lehrer and Gevirtz, 2014). Practical applications in emergency response, military training, and high-stress professions demonstrate that structured reframing techniques—like guided reappraisal and controlled breathing— reduce stress responses. By integrating CBT principles with neurophysiological regulation, reframing becomes a powerful mechanism for transforming threat perception into a challenge-based mindset, fostering mental resilience and long-term psychological growth.

## Tailoring between the available resources and the challenges

Tailoring refers to the process of matching available coping resources to the specific demands of a situation, ensuring that resilience remains dynamic and adaptable rather than a fixed trait. This aligns with Lazarus’s (1984) transactional stress model, which emphasizes evaluating whether a situation requires problem-focused or emotion-focused coping. Resilient individuals do not rely on a single strategy but adjust their coping responses according to contextual demands, supporting effective stress adaptation and psychological well-being.

Neurophysiologically, tailoring involves coordinated activity among the prefrontal cortex (PFC), anterior cingulate cortex (ACC), and hippocampus, which together enable flexible cognitive control (Davidson and McEwen, 2012). The ability to modify coping strategies in real time depends on autonomic regulation, particularly heart rate variability (HRV), which reflects the capacity to shift between stress responses (Lehrer and Gevirtz, 2014). Elevated cortisol levels impair PFC function, resulting in rigid, maladaptive patterns instead of adaptive, tailored coping. In contrast, individuals who regulate physiological arousal effectively tend to show more strategic coping and reduced PTSD risk.

Psychological studies also emphasize flexible coping selection. For example, individuals who use cognitive reappraisal in controllable situations and acceptance-based strategies in uncontrollable ones demonstrate greater emotional stability (Gross, 2015). Training programs that promote flexible coping, decision-making, and autonomic regulation—such as vagal breathing and mindfulness—have shown improved resilience outcomes in both clinical and high-risk groups.

**Figure 1:**
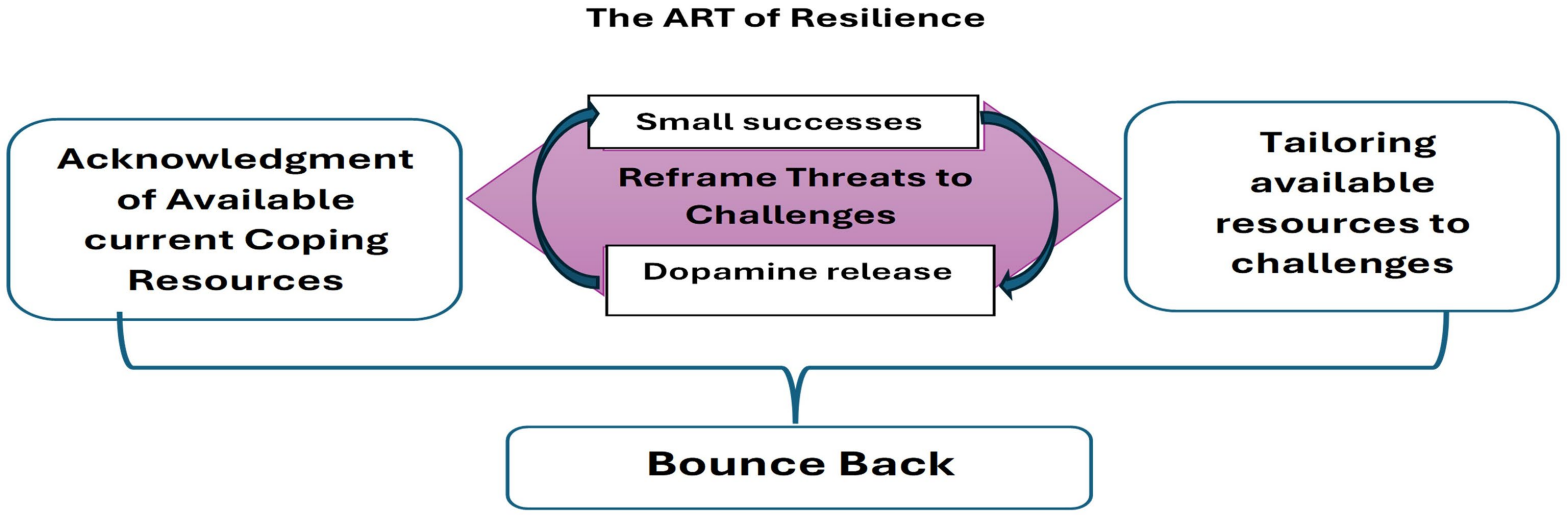
The ART of resilience: The dynamic process.

An illustration of the suggested model is presented in Figure 1.

## Theoretical integration and practical implementation

The evolution of resilience research demonstrates a clear progression from single-perspective approaches to more integrated understandings of how individuals and systems develop and maintain resilient functioning. The ART framework synthesizes these perspectives through its integrated understanding of resilience processes. By incorporating insights from neuroscience and cognitive psychology (Kalisch et al., 2015), the framework demonstrates how seemingly stable traits become dynamically accessible resources during the stress response. Recent intervention studies demonstrate effectiveness of integrated approaches to resilience enhancement (Joyce et al., 2018). Research in developmental resilience (Masten and Cicchetti, 2016) supports the framework’s emphasis on the continuous interaction between individual capabilities and environmental supports. This interaction becomes significant when considering how individuals conserve and utilize resources across different contexts and challenges (Hobfoll, 2001). The framework’s integration of stress mindset research (Crum et al., 2013) further illuminates how cognitive reappraisal processes influence immediate stress responses and long-term adaptive capabilities.

The practical implications of this theoretical integration extend across domains of human functioning. In clinical settings, the framework provides an approach to understanding how individuals can access and deploy their resilience resources effectively. It also offers guidance for developing targeted interventions that consider both personal capabilities and environmental supports. Similarly, the framework’s emphasis on dynamic resource utilization and adaptive response patterns in organizational and educational contexts offers valuable insights for developing systemic approaches to resilience enhancement.

Integrating theoretical perspectives with practical applications represents a significant advancement in resilience research. By demonstrating how different aspects of resilience work together in real-world contexts, the ART framework provides both a theoretical foundation for understanding resilience processes and a practical guide for intervention development. The framework’s emphasis on the dynamic interaction between individual traits, adaptive processes, and environmental supports offers a comprehensive approach to understanding and enhancing resilience across diverse contexts and populations.

## Examples

The ART framework demonstrates its versatility across diverse contexts by focusing on immediate resource utilization and challenge reframing (Hobfoll, 2001; Connor and Davidson, 2003). In community crisis response, basketball teams, or, for example, startups, the process will always begin with identifying currently accessible resources rather than potential ones—reflecting Folkman and Moskowitz's (2000) emphasis on active coping strategies, as implemented in the SIX Cs psychological first aid model (Farchi et al., 2018, 2024). The Recognition phase transforms threats into achievable short-term tasks: communities break down crisis response into specific support actions; basketball teams convert opponent threats into tactical matchup opportunities; and startups segment market challenges into targeted milestones. By creating manageable tasks with a high probability of success, perceived threats are transformed into engaging challenges. The Tailoring phase then matches these accessible resources to immediate challenges—deploying community volunteers effectively, positioning players strategically, or allocating startup resources to critical market opportunities.

## Summary

The ART framework presents a cohesive model for resilience, integrating cognitive, emotional, and contextual components through three interrelated elements: Acknowledgment of available coping resources, Reframing of stressors as challenges, and Tailoring responses to situational demands. Rather than offering a rigid sequence, the model reflects a continuous and flexible process that operates across personal and collective levels. Its added value lies in the emphasis on current, accessible resources and the active adjustment between challenges and coping capacities. As such, ART contributes a practical and theoretically grounded lens for understanding and strengthening resilience.

Beyond its conceptual clarity, the model offers a concrete structure for designing training programs, intervention protocols, and decision-making tools in emergency, clinical, and organizational settings. Its simplicity and applicability make it accessible not only to professionals but also to the general public in real-time contexts.

## Data Availability

The original contributions presented in the study are included in the article/supplementary material, further inquiries can be directed to the corresponding author.
